# Supplementary material for: Innovations in science and scenarios for assessment
Source: Clim Change. 2015 Aug 29;135:55–68. doi: 10.1007/s10584-015-1494-z (PMC4913956; doi:10.1007/s10584-015-1494-z)
Supplement: Supplementary file 1 — (PDF 130 kb) [file 10584_2015_1494_MOESM1_ESM.pdf]

10. References

Bierwagen BG, Theobald DM, Pyke CR, Choate A, Groth P, Thomas JV, and Morefield P (2010) National housing and impervious surface scenarios for integrated climate impact assessments. Proc Natl Acad Sci 107:20887-20892.

Kenneth Kunkel 4/24/2015 3:55 PM
Deleted: et al

Church JA, and White NJ (2011) Sea-level rise from the late 19th to the early 21st Century. Surveys in Geophysics 32:585-602, doi:10.1007/s10712-011-9119-1

Kenneth Kunkel 7/24/2015 9:32 AM
Deleted: Carter LM, Jones JW, Berry L, Burkett V, Murley JF, Obeysekera J, Schramm PJ, and Wear D (2014) Ch. 17: Southeast and the Caribbean. *Climate Change Impacts in the United States: The Third National Climate Assessment*, J. M. Melillo, Terese (T.C.) Richmond, and G. W. Yohe, Eds., U.S. Global Change Research Program, 396-417. doi:10.7930/J0NP22CB.

European Environment Agency (EEA). 2009. Looking Back on Looking Forward: A Review of Evaluative Scenario Literature. European Environment Agency, Copenhagen, Denmark.

Fry J, Xian G, Jin S, Dewitz J, Homer C, Yang L, Barnes C, Herold N, and Wickham J, (2011) Completion of the 2006 National Land Cover Database for the Conterminous United States, PE&RS, 77:858-864.

Hartmann H, Jacobs K, and Moss RH (2012) National Climate Assessment: Scenario Planning Opportunity for Regions and Sectors. Ver: 2012.1.15. Unpublished guidance document distributed through National Climate Assessment Office. See http://www.nesdis.noaa.gov/NCADAC/pdf/nov_16/NCADAC_Mtg_Pres_Nov11_All_Final_111611_5a.pdf for a first draft.

Hayhoe K, and 18 Coauthors (2004) Emission pathways, climate change, and impacts on California. Proc Natl Acad Sci USA 101, 12422-12427.

Kenneth Kunkel 4/24/2015 3:56 PM
Deleted: et al

Hayhoe K and 8 Contributors (2013) Development and Dissemination of a High-Resolution National Climate Change Dataset, 497 ppp. http://cida.usgs.gov/thredds/fileServer/dcp/files/Hayhoe_USGS_downscaled_database_final_report.pdf

Kenneth Kunkel 7/24/2015 9:33 AM
Deleted: Hayhoe K and Coauthors (2008) Regional climate change projections for the Northeast USA. Mitig Adapt Strateg Glob Change 13:425-436.

Kenneth Kunkel 4/24/2015 4:35 PM
Deleted: et al

IPCC (2001) Climate Change (2001) The Scientific Basis. Contribution of Working Group 1 to the Third Assessment Report of the Intergovernmental Panel on Climate Change, edited by Houghton JT, Ding Y, Griggs DJ, Noguer M, van der Linden PJ, Dai X, Maskell K, and Johnson CA (eds). Cambridge University Press, Cambridge, UK, and New York, USA, 2001.

IPCC (2007) Climate Change (2007) The Physical Science Basis. Contribution of Working Group I to the Fourth Assessment Report of the Intergovernmental Panel on Climate Change [Solomon S, Qin D, Manning M, Chen Z, Marquis M, Averyt KB, Tignor M, and Miller HL (eds.)]. Cambridge University Press, Cambridge, United Kingdom and New York, NY, USA.

Janetos AC, Collins W, Wuebbles D, Diffenbaugh N, Hayhoe K, Hibbard K, and Hurtt G (2011) *Climate Change Modeling and Downscaling: Issues and Methodological Perspectives for the U.S. National Climate Assessment.* NCA Report Series, Vol. 7, U.S. Global Change Research Program, 49 pp.

Karl TR, Melillo JT, and Peterson TC, Eds. (2009) *Global Climate Change Impacts in the United States*. Cambridge University Press, 189 pp. [Available online at http://downloads.globalchange. gov/usimpacts/pdfs/climate-impacts-report.pdf]

Keener VW, Hamilton K, Izuka SK, Kunkel KE, Stevens LE, and Sun L (2013) *Regional Climate Trends and Scenarios for the U.S. National Climate Assessment. Part 8. Climate of the Pacific Islands.* NOAA Technical Report NESDIS 142-8, 45 pp.

Kunkel KE, Stevens LE, Stevens SE, Sun L, Janssen E, Wuebbles D, Rennells J, DeGaetano A, and Dobson JG (2013a) *Regional Climate Trends and Scenarios for the U.S. National Climate*

Assessment. Part 1. Climate of the Northeast U.S. NOAA Technical Report NESDIS 142-1, 80 pp.

Kunkel KE, Stevens LE, 12 Coauthors (2013b) *Regional Climate Trends and Scenarios for the U.S. National Climate Assessment. Part 2. Climate of the Southeast U.S.* NOAA Technical Report NESDIS 142-2, 95 pp.

Kunkel KE, Stevens LE, Stevens SE, Sun L, Janssen E, Wuebbles D, Hilberg SD, Timlin MS, Stoecker L, Westcott NE, and Dobson JG (2013c) *Regional Climate Trends and Scenarios for the U.S. National Climate Assessment. Part 3. Climate of the Midwest U.S.* NOAA Technical Report NESDIS 142-3, 96 pp.

Kunkel KE, Stevens LE, 15 Coauthors (2013d) *Regional Climate Trends and Scenarios for the U.S. National Climate Assessment. Part 4. Climate of the U.S. Great Plains*. NOAA Technical Report NESDIS 142-4, 83 pp.

Kunkel KE, Stevens LE, Stevens SE, Sun L, Janssen E, Wuebbles D, Redmond KT, and Dobson JG (2013e) *Regional Climate Trends and Scenarios for the U.S. National Climate Assessment. Part 5. Climate of the Southwest U.S.* NOAA Technical Report NESDIS 142-5, 79 pp.

Kunkel KE, Stevens LE, Stevens SE, Sun L, Janssen E, Wuebbles D, Redmond KT, and Dobson JG (2013f) *Regional Climate Trends and Scenarios for the U.S. National Climate Assessment. Part 6. Climate of the Northwest U.S.* NOAA Technical Report NESDIS 142-6, 76 pp.

Kunkel KE, Stevens LE, Stevens SE, Sun L, Janssen E, Wuebbles D, and Dobson JG (2013g) *Regional Climate Trends and Scenarios for the U.S. National Climate Assessment. Part 9. Climate of the Contiguous United States*. NOAA Technical Report NESDIS 142-9, 78 pp.

Kenneth Kunkel 7/24/2015 9:36 AM

Deleted: Stevens SE, Sun L, Janssen E, Wuebbles D, Konrad II CE, Fuhrman CM, Keim BD, Kruk MC, Billet A, Needham H, Schafer M, and Dobson JG

Kenneth Kunkel 7/24/2015 9:34 AM

Deleted: Stevens SE, Sun L, Janssen E, Wuebbles D, Kruk MC, Thomas DP, Shulski M, Umphlett N, Hubbard K, Robbins K, Romolo L, Akyuz A, Pathak T, Bergantino T, and Dobson JG

MacCracken M, Barron E, Easterling D, Felzer B, and Karl T (2001) Scenarios for climate variability and change. Pages 13-72 in NAST. Climate Change Impacts on the United States. Cambridge University Press, Cambridge, U.K.

Melillo J, Janetos A, Schimel D, and Kittel T (2001) Vegetation and biogeochemical scenarios.in NAST, editor. Climate Change Impacts on the United States: The Potential Consequences of Climate Variability and Change. Cambridge University Press, Cambridge, U.K.

Morgan MG, Cantor R, Clark WC, Fisher A, Jacoby HD, Janetos AC, Kinzig AP, Melillo J, Street RB, and Wilbanks TJ (2005) Learning from the U.S. National Assessment of climate change impacts. Environmental Science and Technology 39:9023-9032.

Moss R, Edmonds JA, and 17 Coauthors (2010) The next generation of scenarios for climate change research and assessment. Nature, 463, 747-756, doi:10.1038/nature08823

Moss RH, Mearns LO, 11 Coauthors (2011) Scenarios for Research and Assessment of Our Climate Future: Issues and Methodological Perspectives for the U.S. National Climate Assessment. NCA Report Series, Volume 6. US Global Change Research Program, Washington, DC. Available at: http://assessment.globalchange.gov

National Climate Assessment Development and Advisory Committee, Ad Hoc Working Group 3, Scenarios and Regional Summaries (2011) Report for NCADAC Meeting May 20, 2011. 37 pp. Available at: http://www.nesdis.noaa.gov/NCADAC/pdf/20b.pdf

NARCCAP, cited 2012: North American Regional Climate Change Assessment Program. [Available online at http://www.narccap.ucar.edu/.

National Assessment Synthesis Team (2000) Climate Change Impacts on the United States: The Potential Consequences of Climate Variability and Change, Report for the US Global

Kenneth Kunkel 4/24/2015 4:37 PM
Deleted: et al

Kenneth Kunkel 4/24/2015 4:38 PM
Deleted: et al

Change Research Program, 163 pp., U.S. Global Climate Research Program, Washington, DC. Available online at: http://library.globalchange.gov/downloads/download.php?id=124

National Research Council (NRC) (2007) *Analysis of Global Change Assessments: Lessons Learned*. National Research Council, Committee on Analysis of Global Change Assessments, Board on Atmospheric Sciences and Climate, Division on Earth and Life Studies. National Academies Press, 196 pp.

National Research Council (NRC) (2009) Informing decisions in a changing climate. Panel on Strategies and Methods for Climate-Related Decision Support. Washington, DC: The National Academies Press.

National Research Council (2012). Sea-Level Rise for the Coasts of California, Oregon, and Washington: Past, Present, and Future. Washington, DC: The National Academies Press.

Parris AP, Bromirski P, Burkett V, Cayan D, Culver M, Hall J, Horton R, Knuuti K, Moss R, Obeysekera J, Sallenger A, and Weiss J (2012) Global Sea Level Rise Scenarios for the US National Climate Assessment. NOAA Tech Memo OAR CPO-1. 37 pp.

Parson EA, Morgan MG, Janetos A, Joyce L, Miller B, Richels R, and Wilbanks TJ (2001) The socioeconomic context for climate impact assessment. Pages 93-107 in NAST, editor. Climate Change Impacts on the United States: The Potential Consequences of Climate Variability and Change. Cambridge University Press, Cambridge, U.K.

Rahmstorf S (2007) A semi-empirical approach to projecting future sea-level rise, Science, 315:368-370, DOI: 10.1126/science.1135456.

Kenneth Kunkel 7/24/2015 9:36 AM
Deleted: ... [1]

Salter J, Robinson J., Wiek A (2010) Participatory methods of integrated assessment—a review. Wiley Interdisciplinary Review Climate Change 1, 697-717.

Stewart BC, Kunkel KE, Stevens LE, Sun L, and Walsh JE (2013) *Regional Climate Trends and Scenarios for the U.S. National Climate Assessment. Part 7. Climate of Alaska*. NOAA Technical Report NESDIS 142-7, 61 pp.

Van den Broeke MR, Bamber J, Lanaerts J, and Rignot E (2011) Ice sheets and sea level: thinking outside the box. Surv Geophys. 32:495-505, DOI 10.1007/s10712-011-9137-Z

Wood A, Maurer E, Kumar A, and Lettenmaier D (2002) Long-range experimental hydrologic forecasting for the eastern United States. J Geophys Res 107 Art. No. 4429.

Kenneth Kunkel 7/24/2015 9:36 AM

Deleted: Vermeer M and Rahmstorf S (2009) Global sea level linked to global temperature. Proc Natl Acad Sci, 106:21527–21532, doi:10.1073/pnas.0907765106.
