# Supplementary material for: Innovations in science and scenarios for assessment
Source: Clim Change. 2015 Aug 29;135:55–68. doi: 10.1007/s10584-015-1494-z (PMC4913956; doi:10.1007/s10584-015-1494-z)
Supplement: Supplementary file 2 — (PDF 361 kb) [file 10584_2015_1494_MOESM2_ESM.pdf]

Innovations in Science and Scenarios for Assessment

Kenneth E. Kunkel[1]

Cooperative Institute for Climate and Satellites

North Carolina State U. and NOAA National Climatic Data Center

Richard Moss

University of Maryland

Adam Parris

Climate Program Office

National Oceanic and Atmospheric Administration

submitted to *Climatic Change*

[1] Corresponding author address: National Climatic Data Center, 151 Patton Avenue, Asheville, NC 28801; ken.kunkel@noaa.gov; phone: (828) 257-3137; fax: (828) 257-3002

Abstract

For the Third National Climate Assessment (NCA3), scenarios were produced for physical climate and sea level rise with substantial input from disciplinary and regional experts. These scenarios underwent extensive review and were published as NOAA Technical Reports. For land use/cover and socioeconomic conditions, scenarios already developed by other agencies were specified for use in the NCA3. Efforts to enhance participatory scenario planning as an assessment activity were pursued, but with limited success. Issues and challenges included the timing of availability of scenarios, the need for guidance in use of scenarios, the need for approaches to nest information within multiple scales and sectors, engagement and collaboration of end users in scenario development, and development of integrated scenarios. Future assessments would benefit from an earlier start to scenarios development, the provision of training in addition to guidance documents, new and flexible approaches for nesting information, ongoing engagement and advice from both scientific and end user communities, and the development of consistent and integrated scenarios.

Kenneth Kunkel 7/28/2015 3:00 PM
Deleted: identified and

1. Introduction

The US National Climate Assessment's (NCA) need for observations and future scenarios of socioeconomic, climate, and environmental conditions arises from requirements in the 1990 Global Change Research Act (GCRA). Specifically, in preparing and submitting an assessment to the President and Congress, the report should, among other things, "analyze current trends in global change, both human-induced and natural, and project major trends for the subsequent 25 to 100 years" (GCRA, Section 106). Responding to this requirement, the Third NCA (NCA3)

Kenneth Kunkel 7/27/2015 7:39 PM
Deleted: set forth

undertook an ambitious effort to provide new information on past and potential future conditions for the assessment process. The scenario effort was planned by a working group of the National Climate Assessment Development and Advisory Committee (NCADAC). Implementation drew on the resources and expertise of the US Global Change Research Program (USGCRP), the National Oceanographic and Atmospheric Administration (NOAA), the National Aeronautics and Space Administration, the Environmental Protection Agency (EPA), the Department of Energy (DOE), the Department of the Interior, and other agencies. A technical support unit (TSU) at the National Climatic Data Center prepared climate information, while other components of the scenarios drew on a variety of existing resources published in the peer-reviewed research literature. A key challenge was attempting to move beyond traditional scenarios approaches, focusing instead on facilitating consistency and coordination among those producing the assessment and using scenarios to facilitate communication with end users. Innovations included a set of regional climate outlooks that provided narrative descriptions of past and potential future climate features of importance to assessors and potential end users, a range of sea level change scenarios considering multiple potential users, and resources to support participatory scenario planning as a way to better engage decision makers, resource managers, and communities.

Kenneth Kunkel 4/24/2015 10:27 AM
Deleted: of several agencies participating in

Kenneth Kunkel 4/24/2015 10:27 AM
Deleted: including

Kenneth Kunkel 7/27/2015 7:41 PM
Deleted: for the scenario effort

The authors of this paper were all involved in various aspects of the scenario process, so this is not a disinterested evaluation of what was planned and achieved. Rather, our purpose is to describe NCA3 scenario objectives and products, including several innovations from prior NCAs, and to provide our reflections on topics for evaluation and additional innovations for future scenario and assessment planners to consider. We also seek to advance the research

literature on preparation and use of multi-component global change scenarios for research and assessment purposes.

We first briefly review the scenarios provided in the first two NCAs, and describe the strategy of the NCADAC's scenario working group (SWG). We then review in detail the climate, sea level, integrated socioeconomic and land cover/land use, and scenario planning components of the scenario strategy. We conclude with questions and issues to be considered for the sustained national assessment and future assessment reports.

2. Scenarios in the 2000 and 2009 NCA reports

The 2000 assessment (NAST, 2000) and 2009 report (Karl et al., 2009) were very different in character, which is reflected in their scenarios (Moss et al., 2011). The 2000 NCA was similar to NCA3 in that one of its objectives was to establish networks of assessors and end users that would persist following report completion. Another similarity was its level of ambition: multiple products including multiple regional and sectoral chapters and a range of summary products. Three basic scenarios types were provided: climate (MacCracken et al., 2001); ecosystems/vegetation (Melillo et al., 2001); and socioeconomic (Parson et al., 2001). Selection of climate scenarios was based on availability of data at the time of the assessment. The scenarios were based on two Global Climate Models (GCMs): the U.K. Hadley Model (HadCM2) and the Canadian GCM (CGCM1). Only one emissions scenario was used to 'force' (i.e., as an exogenous input to) the models: the Intergovernmental Panel on Climate Change (IPCC) IS92a (a mid-range 'business as usual' emissions future). Some climate diversity was provided because HadCM2 was relatively cooler and wetter and CGCM1 warmer and drier. Controversy arose because no US models were represented and the use of only one emissions

Kenneth Kunkel 4/23/2015 8:17 AM
Deleted: dimensional

Kenneth Kunkel 7/28/2015 3:01 PM
Deleted: planned by

Kenneth Kunkel 4/24/2015 11:50 AM
Deleted: 1

Richard Moss 7/26/2015 2:22 PM
Deleted: 0

Kenneth Kunkel 7/24/2015 2:08 PM
Deleted: developed for each

Kenneth Kunkel 7/27/2015 7:43 PM
Deleted: the

Kenneth Kunkel 7/27/2015 7:44 PM
Deleted: of the report

Kenneth Kunkel 7/24/2015 2:08 PM
Deleted: were produced

Kenneth Kunkel 7/8/2015 9:45 AM
Deleted: C

Kenneth Kunkel 7/8/2015 9:46 AM
Deleted: , but

Kenneth Kunkel 7/8/2015 9:46 AM
Deleted: c

future did nothing to bound the true uncertainty (Morgan et al., 2005). Terrestrial ecosystem/vegetation scenarios were produced through the Vegetation-Ecosystem Modeling and Analysis Project (VEMAP) to provide information on ecosystem shifts resulting from climate change. Socioeconomic projections for a small number of population and economic variables at county scale to 2030 and aggregate national scale projections from 2030 to 2100 were provided from an integrated assessment model. Guidance was provided for assessment authors to develop simple parametric projections of variables of interest for the assessment of impacts in particular places or sectors. According to Morgan et al. (2005), the socioeconomic scenarios were not widely used, and most participants suggested another approach was needed for the future.

By contrast, the 2009 assessment needed to be completed in a short period of time (13 months) to comply with a legal ruling that the "Synthesis and Assessment Products" prepared by the program on a variety of climate science and impacts topics failed to fulfill the requirements of the GCRA. The resulting assessment focused on integrating information contained in these reports, supplemented with more recent findings in some areas. Report authors used climate information based on a set of fifteen models from the Coupled-Model Intercomparison Project Phase 3 (CMIP3) forced by the IPCC Special Report on Emissions Scenarios (SRES) B1 (low emissions) and A2 (high emissions) scenarios. Many of these models were statistically downscaled to a higher spatial and temporal resolution using a bias-corrected spatial disaggregation (BCSD) method (Wood et al. 2002). The statistical downscaled data were used to produce several derived climate variables (e.g., number of days > 90°F). A variety of

Richard Moss 7/26/2015 2:18 PM
Deleted: scenarios were quantitative

Richard Moss 7/26/2015 2:19 PM
Deleted: produced using a commercial regional economic growth model, and

Richard Moss 7/26/2015 2:19 PM
Deleted: for aggregate national scale projections from 2030 to 2100

Kenneth Kunkel 7/8/2015 9:47 AM
Deleted: was primarily focused on completing a report

Kenneth Kunkel 7/8/2015 9:48 AM
Deleted: related to failure

Kenneth Kunkel 7/8/2015 9:48 AM
Deleted: to complete an assessment every four years

Kenneth Kunkel 7/8/2015 9:49 AM
Deleted: Cl

Richard Moss 7/26/2015 2:20 PM
Deleted: was

Kenneth Kunkel 4/24/2015 10:44 AM
Deleted: #

specialized maps, projections, and indicators were produced using these data and appeared in the report. No socioeconomic scenarios were provided.

3. Overview of NCA3 scenario strategy and innovation

NCA3 scenarios preparation began with workshops in 2010 on scenarios (Moss et al, 2011) and modeling (Janetos et al., 2011). These engaged a broad range of researchers, intermediate users (mostly climate modelers and impacts scientists), end users, and USGCRP agency research program managers. The scenarios workshop focused on types of scenarios (climate, socioeconomic, etc.) and their inter-relationships, reviewed previous scenario experience in the NCA and other assessments, provided an overview of ongoing scenario efforts, and included extensive discussion of user needs. Climate, socioeconomic, and user support community discussions were also held to plan scenario material for the NCA3.

Development of a detailed scenario strategy fell to the SWG. The group included approximately 20 NCADAC members and representatives from several federal agencies, incorporating wide research expertise, individuals from the private sector, and users. The group met virtually over a roughly three-month period and developed recommendations that were presented to the NCADAC in May, 2011 (NCADAC Ad Hoc Working Group on Scenarios, 2011) and approved in November 2011. The SWG made five sets of specific recommendations on historical observations and future scenario information for: 1) climate, including traditionally-provided global scenarios, regional downscaling, and narrative climate 'outlooks'; 2) global sea level rise including overview of factors that result in regional anomalies; 3) land cover and land use; 4) socioeconomic conditions; and 5) participatory scenario planning activities. Each of

Richard Moss 7/26/2015 2:21 PM
Deleted: information was

Kenneth Kunkel 7/28/2015 3:03 PM
Deleted: , both held in 2010

Richard Moss 7/26/2015 2:23 PM
Deleted: from regional, sectoral, and resource management perspectives in a series of breakout groups and plenary discussions

Richard Moss 7/26/2015 2:24 PM
Deleted: begin planning

Richard Moss 7/26/2015 2:26 PM
Deleted: (including some ex-officio members

Richard Moss 7/26/2015 2:26 PM
Deleted:)

Richard Moss 7/26/2015 2:26 PM
Deleted: from the

Richard Moss 7/26/2015 2:29 PM
Deleted: climate science, socioeconomic, ocean/coastal sciences, and biophysical research

Richard Moss 7/26/2015 2:26 PM
Deleted: communities

Richard Moss 7/26/2015 2:29 PM
Deleted: ,

Richard Moss 7/26/2015 2:29 PM
Deleted: other

Richard Moss 7/26/2015 2:29 PM
Deleted: users, including Native American user groups

Richard Moss 7/26/2015 2:30 PM
Deleted: and

Richard Moss 7/26/2015 2:30 PM
Deleted: scenarios

Richard Moss 7/26/2015 2:31 PM
Deleted: piloting

these topics is discussed in greater detail below, but several general points about the SWG's objectives are relevant.

The strategy considered needs for two broad categories of users: scenarios for intermediate users, especially to support and coordinate modeling and synthesis; and scenarios and related tools intended to communicate with lay audiences and to support participatory processes considering the implications of climate change. There is some overlap in these two sets of needs, but there are also important tensions. For example, intermediate users wanted consistent scenarios reflecting scientific consensus on the range of plausible outcomes, whereas risk managers would be better served by scenarios that take into account low probability, high consequence events (potential surprises) (EEA, 2009).

The more extensive and diverse set of products envisioned by the SWG was intended to address unmet needs noted by authors of prior assessments (mostly intermediate users) and to provide new types of resources that communicated more effectively with end users. This latter use was seen as supporting the shift to a sustained assessment process based on ongoing interactions between the research and user communities.

One of the most important innovations was the decision to produce 'regional climate outlooks', which included narrative descriptions customized for each region. This approach allowed for comparing and discussing complex scientific results in a more accessible fashion than if only model data sets were provided, thus fulfilling the objective of meeting needs of end users and those intermediate users who looked to scenarios for 'context'. The outlooks provided both historical trends of several climatological variables including means and extremes and discussion of current understanding of the future, based on simulations forced by

Richard Moss 7/26/2015 2:33 PM
Deleted: in a range of decision and deliberative settings

Richard Moss 7/26/2015 2:36 PM
Deleted: the former set would benefit from

Richard Moss 7/26/2015 2:37 PM
Deleted: that represent a

Richard Moss 7/26/2015 2:33 PM
Deleted: more likely

Richard Moss 7/26/2015 2:39 PM
Deleted: the latter's interest in exploring risk management issues that arise from

Richard Moss 7/26/2015 2:39 PM
Deleted: low probability, high consequence events would be better served withprofit from a diversity of scenarios

Kenneth Kunkel 7/24/2015 2:41 PM
Deleted: of the state of understanding of climate

Richard Moss 7/26/2015 2:40 PM
Deleted: to some degree

Richard Moss 7/26/2015 2:41 PM
Deleted: included

Kenneth Kunkel 4/24/2015 3:24 PM
Deleted: climatologi

Kenneth Kunkel 4/24/2015 11:05 AM
Deleted: es

Richard Moss 7/26/2015 2:42 PM
Deleted: "

the A2 (high) and B1 (low) emissions scenarios (Appendix 1, NCADAC Ad Hoc Working Group on Scenarios, 2011). These scenarios were selected because they bounded uncertainty about future emissions and socioeconomic conditions and because a large body of impacts research was based on them, facilitating preparation of report chapters.

A second innovation was inclusion of sea level change scenarios for risk framing. Sea level scenarios had not been included in prior assessments, but the increasing visibility of coastal vulnerabilities and need for information about potential future conditions for planning purposes created a strong demand. For risk framing purposes, large uncertainty bounds were selected to provide users with high-consequence but scientifically grounded futures. The scenarios covered global mean changes to 2100 and descriptions of the factors that cause regional variations.

The third major advance was preparation of guidelines and materials to support more widespread experimentation with participatory scenario planning. The primary purpose of participatory scenario processes is to identify strategies and make decisions robust to a wide range of future conditions. The idea was to encourage and support lead author teams who expressed interest in using such techniques in workshops and stakeholder engagements associated with preparation of their chapters.

4. Description and Innovations in Physical Climate Information

Two groups provided advice on physical climate information: (1) the SWG; and (2) a selected group of climate model experts (CMEs). There was considerable discussion about whether to use CMIP3 or CMIP5 model simulations. Although the CMIP5 archive represented the latest set of coordinated GCM simulations, the primary recommendation was to use the

Kenneth Kunkel 7/28/2015 3:04 PM
Deleted: .

Kenneth Kunkel 7/24/2015 2:44 PM
Deleted: as the primary basis for the high climate future

Richard Moss 7/26/2015 2:41 PM
Deleted: … and by the B1 (low) emissions scenario as the primary basis for the low climate future…" for climate scenarios

Richard Moss 7/26/2015 2:43 PM
Deleted: a very

Richard Moss 7/26/2015 2:43 PM
Deleted: set of

Richard Moss 7/26/2015 2:44 PM
Deleted: potential robust options

Richard Moss 7/26/2015 2:44 PM
Deleted: for development, resource and land management, and other activities

Kenneth Kunkel 7/24/2015 11:26 AM
Deleted: for use by NCA3 authors

Kenneth Kunkel 7/24/2015 11:27 AM
Deleted: T

CMIP3 simulations, largely because it was uncertain whether a full set of the CMIP5 simulations could be made available in a timely manner to the NCA3 chapter authors and whether any differences between the simulations would be significant enough to alter key conclusions in the NCA evaluation of impacts. A secondary recommendation was to allow the use of CMIP5 simulations as time and resources permitted, specifically the Representative Concentration Pathways (RCP) 2.6 and 8.5 scenarios, as representing a wider range of outcomes than the CMIP3 scenarios. The low emissions B1 scenario in the SRES family is most comparable to RCP4.5, not the lowest in the RCP family. The lowest, RCP2.6, represents a more stringent mitigation scenario than the previous low-end scenarios and provides a larger decision space for consideration. The NCADAC considered these recommendations and decided to use scenarios based on the CMIP3 A2 and B1 simulations as the primary ones that authors were asked to consider, while also allowing use of CMIP5 simulations for illustrative purposes.

The development of regional outlooks involved about 30 regional experts in addition to climate scientists from NCDC. A common set of information was produced for all of the regions. This information was disseminated in the form of a NOAA NESDIS Technical Report series (NOAA NESDIS TR142; Kunkel et al. 2013a,b,c,d,e,f,g; Stewart et al. 2013; Keener et al. 2013). This series included over 700 pages of material and several hundred graphics on historical trends and future projections. Several of the analyses and graphics documented climate model projection uncertainty.

Many regional experts were involved as technical report authors. This enriched the historical material and insured the inclusion of important elements. Many analytic and graphical depictions were common across the regions, providing a level of uniformity not

Kenneth Kunkel 7/24/2015 11:27 AM
Deleted: . However,

Kenneth Kunkel 7/24/2015 11:28 AM
Deleted: In addition, the CMIP3 simulations had been exhaustively analyzed and vetted, a process just beginning for CMIP5. ... [1]

Kenneth Kunkel 4/24/2015 3:26 PM
Deleted: RCP

Kenneth Kunkel 7/24/2015 11:29 AM
Deleted: Each report consisted of two main sections: one on historical conditions and trends, the other on future conditions as simulated by CMIP3 models and associated downscaled data sets. The reports concentrated on temperature and precipitation.

Kenneth Kunkel 7/24/2015 11:29 AM
Deleted: There were several unique features of this information for NCA3 authors.

Kenneth Kunkel 7/24/2015 11:29 AM
Deleted: , primarily contributing to the historical sections

Richard Moss 7/26/2015 3:39 PM
Deleted: sis

available in previous assessments. The reports were subjected to an intensive anonymous review process involving about 20 reviewers.

The projection information in TR142 utilized three sources of data—climate model data for 15 CMIP3 models and two downscaled data sets: a statistically-downscaled data set by Hayhoe et al. (2004; 2008) and the dynamically-downscaled data set from the North American Regional Climate Change Assessment Program (NARCCAP 2012). The dynamic (NARCCAP) and statistical downscaled data sets provide better representation of spatial patterns in areas of high topographic variability and, in the case of statistically downscaled data sets, biases are lower because of the bias correction methods used in their development. These higher resolution data sets were used to develop the projection products for derived daily resolution impacts relevant climate variables, such as threshold exceedance number. Because of their advantages over GCM data (better representation of spatial patterns, bias correction), these downscaled data sets were the primary basis for developing projections of metrics of extremes.

The projection information included presentations of both mean changes and model spread as one indicator of model uncertainty. The interpretation and communication of model spread/uncertainty was an area of focus for the NCA3. For temperature projections, there is a substantial model spread (a factor of 2-3 difference among models), but the multi-model mean warming is very large with respect to historical variations. Thus, unequivocal statements were made about large future warming. Precipitation projections were a very different case because future changes are small and not statistically significant for large portions of the U.S. However, this is largely due to the geographic position of the contiguous U.S., straddling the transition zone between projected wetter conditions at higher mid-latitudes and drier conditions in the

Kenneth Kunkel 7/24/2015 11:30 AM
Deleted: The NOAA Technical Report (TR142) included maps of the CMIP3 and NARCCAP analyses and tabular summaries of the statistically-downscaled data.

Kenneth Kunkel 7/28/2015 3:06 PM
Deleted: were included because their higher resolution was believed to be characterized by lower biases at the regional and local scales of interest to the NCA, and in particular to

Kenneth Kunkel 7/24/2015 11:30 AM
Deleted: Analysis of these downscaled data sets was performed in consultation with the providers to insure proper usage.

subtropical belts. This context was viewed as important in communicating the small projected changes.

About the time that TR142 was published and the public review draft of the NCA3 released, a new statistically-downscaled data set became available (Hayhoe et al. 2013). This data set uses more sophisticated methods for daily downscaling, more suitable for extremes analysis. In particular, the newer method downscales from daily GCM data, thus preserving the daily sequence of weather conditions in the GCM simulation and allowing for sub-monthly temporal patterns outside the range of historical conditions, whereas the older method produces daily resolution data by randomly sampling historical months. Although it was too late to incorporate this newer data set into TR142, all NCA3 figures generated using the older Hayhoe et al. (2004) data set were regenerated using this new data set.

Reflections and issues for evaluation and planning

As CMIP5 data availability increased, it became clear that a basic analysis of CMIP5 data could be done in time for NCA3, although not in time for any impacts research that would support the work of the regional and sectoral chapter authors. Differences arose within the climate science author team about the emphasis on CMIP3 vs CMIP5, with some authors contending that CMIP3 data were too dated for inclusion, despite the NCADAC decision. A compromise was reached to include a series of graphics in the climate science sections to compare CMIP3 and CMIP5 results.

At the center of this issue was the relatively high level of autonomy of the chapter author teams, combined with their lack of involvement in upfront decisions about content in this critical chapter. The overlap among the advisory groups, author teams, and NCADAC

Kenneth Kunkel 7/24/2015 11:31 AM
Deleted: ... [2]

Kenneth Kunkel 4/24/2015 3:28 PM
Deleted: A

Kenneth Kunkel 7/24/2015 11:31 AM
Deleted: , 2008

Kenneth Kunkel 7/24/2015 3:12 PM
Deleted: time progressed and

Richard Moss 7/26/2015 2:49 PM
Deleted: in-depth impacts analysis

Richard Moss 7/26/2015 2:50 PM
Deleted: depicting future projections for both

decision-making body was relatively small. Earlier selection of author teams and their upfront involvement in decisions could help avoid such conflicts, though there will always be challenges associated with evolving state of knowledge during assessment processes.

There were requests from authors for historical trend and future scenario information on variables other than temperature and precipitation (e.g. wind and solar energy). This need was recognized from the beginning of scenarios development but the time needed for spin-up of capacity limited the scope of analysis. Future assessments may benefit from a sustained assessment process that maintains capabilities across assessment cycles.

Managing the involvement of a large group of regional experts in developing regional outlooks was time-consuming. However, the quality of this group's contributions made this worthwhile. It ensured that regional issues of importance were not overlooked and that the most relevant regional research and analyses were incorporated. For example, information about Lake Champlain ice cover was added to the Northeast Technical Report (Kunkel et al. 2013a) by regional experts and also incorporated into the NCA3 (Appendix 3).

5. Sea level change: a new frontier for the NCA

Evidence for global mean SLR has been increasingly documented in assessments of the Intergovernmental Panel on Climate Change (IPCC), the National Research Council (NRC), and a growing body of peer-reviewed scientific literature (IPCC 2001, IPCC 2007, Church and White 2011), although the IPCC reports received extensive criticism about the implications of ice melt (e.g. Rahmstorf 2007, Van den Broeke et al. 2011). The IPCC Fifth Assessment Report also includes projections of global mean SLR and the National Research Council published a report on projected SLR in California, Oregon, and Washington in 2012 (NRC 2012). State and local

Kenneth Kunkel 7/24/2015 3:10 PM
Deleted: in the case of the climate science chapter, with only one NCADAC member as an author

Kenneth Kunkel 7/24/2015 3:11 PM
Deleted: The physical climate information in TR142 was largely concentrated on temperature and precipitation.

Kenneth Kunkel 7/24/2015 3:11 PM
Deleted: other

Kenneth Kunkel 7/24/2015 3:12 PM
Deleted: very

Kenneth Kunkel 7/24/2015 2:46 PM
Deleted: a very cost-effective dimension

Kenneth Kunkel 4/24/2015 3:29 PM
Deleted: et al.

Kenneth Kunkel 7/24/2015 2:46 PM
Deleted: Rignot et al. 2004,

Kenneth Kunkel 7/24/2015 2:47 PM
Deleted: Vermeer and Rahmstorf 2009,

Kenneth Kunkel 4/24/2015 3:30 PM
Deleted: 3

governments and the US Army Corps of Engineers also are developing or have already developed their own SLR projections for coastal planning, policy, and management from the existing body of scientific literature (Parris et al 2012). However, prior to the third NCA, a similar process had not been done at the national scale in an interagency context.

The NCADAC requested an assessment of the scientific literature on global SLR, primarily to help author teams use a consistent approach to projected impacts from future global SLR. A diverse group of experts in science, engineering, and coastal management convened to identify the scenarios. The group was a mix of members from five different federal agencies, eight different academic institutions, and a regional government agency. Thus, the identification of scenarios and synthesis of scientific literature was grounded in rich and diverse experience and expertise, including national coastal risk reduction measures, national coastal management and flood policy, local coastal management policies and problems, and fundamental science on sea level rise and coastal flooding.

Two dimensions of this work proved to be critical. First, for both practical and scientific reasons, the author team consulted with the NCADAC and decided to identify global, rather than regional, scenarios, while still providing a summary of the literature on important processes leading to regional and local variations of sea level change. The agreed-upon goal was scenarios to bound global conditions within which assessment authors and regional and local experts could conduct more context specific analyses.

Second, the authors embraced an integrated body of social and behavioral science and risk management practice focused on decision making under uncertainty. Specifically, they examined and identified a broad range of scenarios to support preparedness for a range of

Kenneth Kunkel 7/8/2015 9:52 AM
Deleted: synthesis

Adam Parris 7/27/2015 2:58 PM
Deleted: an innovative

Kenneth Kunkel 7/24/2015 2:48 PM
Deleted: focused on water management in Southwest Florida

Adam Parris 7/27/2015 2:58 PM
Deleted: Additionally, three of the federal experts who authored the report had extensive experience in working with or for regional and local coastal management programs.

Kenneth Kunkel 7/24/2015 2:48 PM
Deleted: the theory and process of

Adam Parris 7/27/2015 3:10 PM
Deleted: decided, in consultation

Adam Parris 7/27/2015 3:10 PM
Deleted: ,

Richard Moss 7/26/2015 2:59 PM
Deleted: Technical input reports compiled for the NCADAC were being written contemporaneously, including both a sectoral report on coasts and multiple regional analyses that included coastal areas. These reports included input from a more diverse array of experts, and it would have been impossible and inefficient to try to coordinate all of those teams in real time. Therefore, the agreed-upon goal was to produce a report providing scenarios to bound global conditions, within which regional and local experts

future conditions. Each scientifically-rigorous scenario was based on plausible future conditions of the Earth system, and on input from managers regarding various coastal management contexts.

The scenarios are bounded by a high-end estimate of global sea level rise by 2100 (6.6 feet or 2 meters) and a low-end estimate (8 inches or 0.2 meters), with two intermediate scenarios. Scenarios were based on multiple scientific methods: extension of historically observed trends, projections from global climate models, semi-empirical models, and empirical calculations of hypothetical conditions. An additional decision was made not to assign likelihoods or confidence statements to individual scenarios, but to assess confidence in the full range of possible sea level rise by the end of the century. The result was the statement:

> "We have very high confidence (>9 in 10 chance) that global mean sea level will rise at least 0.2 meters (8 inches) and no more than 2.0 meters (6.6 feet) by 2100"

While the middle of this range captured by the intermediate scenarios (1 to 4 feet) may be considered more likely, a narrower range also would be necessarily assigned less confidence because peer-reviewed evidence indicates higher amounts of sea level rise are possible by the end of the century. The wide range was intended to support risk management applications. The lowest, intermediate-low, and intermediate-high can all be logically connected to the A2 or B1 emissions scenarios.

Reflections and issues for evaluation and planning

While intended for use by NCA authors as a foundational document, report preparation extended well into the development of the NCA draft. Input from the NCADAC and peer reviewers improved the document to the point where federal agencies and stakeholders of the

Kenneth Kunkel 7/27/2015 7:57 PM
Deleted: the end of the century

Richard Moss 7/26/2015 3:05 PM
Deleted: climate

Richard Moss 7/26/2015 3:06 PM
Deleted: The authors wanted to communicate an expected range of sea level rise with very high confidence.

Richard Moss 7/26/2015 3:07 PM
Deleted: , as the literature summarized in the report reveals, there is notable,

Richard Moss 7/26/2015 3:07 PM
Deleted: that

NCA perceived utility in providing it to the public for other applications. To date, one of the most prominent uses of the report have been support for the President's Superstorm Sandy Recovery Task Force and national coastal flood risk policy deliberation.

The decision to base the scenarios on multiple scientific methods complicated the justification for each scenario, but is an important innovation. The climate modeling community emphasizes the importance of using an ensemble approach to hedge against the bias of any one model. It is equally important to consider different types of scientific analyses outside of models. Where estimates from different methods or different forms of evidence converge, we can better assess our confidence in estimates of future change.

The risk-based framing proved a useful scenario planning approach, while also addressing NCADAC guidance regarding use of the A2 and B1 emissions scenarios. The Lowest Scenario (0.2 m) is a linear extrapolation of the observed 20th century trend, which could occur under a B1 or RCP2.6 emissions pathway. These pathways require very near-term implementation of aggressive strategies to reduce greenhouse gas emissions – a point explicitly stated in the report. Some authors felt strongly that extrapolation of observed trends provided a rational baseline for a low-end scenario to be compared to model projections. However, the risk-based framing helped distinguish that the Lowest Scenario should only be used where there is a very high tolerance of risk.

6. Land cover/use and socioeconomic scenarios

NCA3 products and approach

Land cover influences carbon sequestration, regulates water flows and quality, and affects climate through numerous feedback mechanisms (e.g., release of greenhouse gases

Richard Moss 7/26/2015 3:11 PM
Deleted: T

Kenneth Kunkel 7/28/2015 3:12 PM
Formatted: Indent: First line: 0.5"

Kenneth Kunkel 7/27/2015 7:58 PM
Deleted: The decision to base the scenarios on multiple scientific methods complicated the justification for each scenario, but is an important innovation. The climate modeling community emphasizes the importance of using an ensemble approach to hedge against the bias of any one model. It is equally important to consider different types of scientific analyses outside of models. Where estimates from different methods or different forms of evidence converge, we can better assess our confidence in estimates of future change.

from land cover changes such as sudden forest dieback or melting of permafrost). Land-based resources support forestry and agriculture products whose production can be affected by changes in climate. Land cover and use in the United States are influenced by numerous factors including climate, but others as well, such as demand for forest and agricultural products, population growth and migration, urbanization, and environmental regulations. Significant changes have occurred over the past several decades, including urban growth, increases in forested areas, and land area reductions for agricultural production.

Numerous government agencies use a variety of technologies for monitoring ongoing changes in land *cover*, including satellite and *in situ* measurements. Land *use* patterns are more difficult to monitor, and sources of data include those for land cover, plus statistical data associated with planning, zoning, and other environmental and development policy processes. The wide array of data and products became a challenge for the preparation of land cover and use data and scenarios for NCA3, especially in the context of the NCADAC SWG's lack of information regarding the needs of different chapter author teams and potential end users.

Kenneth Kunkel 4/24/2015 3:31 PM
Formatted: Font:Italic

Richard Moss 7/26/2015 3:03 PM
Deleted: uncertainty

Richard Moss 7/26/2015 3:03 PM
Deleted: information

The SWG did not converge on a grand design or integrated approach for land cover information, but instead recommended two existing peer-reviewed sources. For baseline land cover data, the National Land Cover Database (NLCD) 2006 (Fry et al., 2011) allocates land cover into 16 different classes and has been applied consistently across all 50 states and Puerto Rico at a spatial resolution of 30 meters. For future scenarios of land cover and use, the EPA Global Change Research Program's Integrated Climate and Land Use Scenarios (ICLUS) Project provided downscaled population and land use scenarios based on global SRES scenarios, including B1 (low) and A2 (high) emissions futures (see http://www.epa.gov/global-

adaptation/iclus/nca_regions.html). Their consistency with the climate scenarios and information was one of the factors that supported their use. The scenarios present information on housing density and impervious surface cover that are consistent with the SRES storylines (Bierwagen et al., 2010).

Reflections and issues for evaluation and planning

Given the short timelines and lack of consensus on how to produce land cover/use and socioeconomic scenarios, it was fortunate that the existing NLCD and ICLUS products met the minimum NCADAC requirements. But the information provided was most likely less than adequate given the diverse needs across the wide range of ecological and socioeconomic topics covered in the NCA. For example, socioeconomic vulnerabilities to climate change depend in large part on where people are and where they are migrating (demography), what their livelihoods are (economic patterns and changes), how they regulate business and govern daily activities (institutions), what cultural and social patterns influence behavior, and what adaptation options are available (e.g., technologies, management systems, and social networks). Unlike the climate and sea level scenarios, none of the agencies responsible for more operational activities, such as the US Census Bureau and the Bureau of Economic Analysis, were in a position to follow up on their initial interest in supporting the activity. Thus it fell to a small subset of the SWG, with support from the NCA office, to implement the strategy using existing products.

It is unclear how the land cover/use and socioeconomic scenarios were used, if at all. Contributing factors likely included diversity of needs, less familiarity with application of these scenarios, lack of support, and time pressure on author teams. These speculations

Kenneth Kunkel 4/24/2015 3:32 PM
Deleted: , Theobald,

Kenneth Kunkel 7/8/2015 9:54 AM
Deleted: that

Kenneth Kunkel 7/8/2015 9:54 AM
Deleted: , the information provided was most likely less than adequate

notwithstanding, development of a better strategy to support the sustained assessment and preparation of future assessment reports will require evaluation of the use of this information in NCA3 and an analysis of the opportunity cost of providing this kind of information.

7. Participatory scenario planning: exploring integration of climate science and applications

NCA3's pilot approach

The SWG, responding to increased experience with participatory approaches that mix experts and end users in assessment and decision support processes (NRC, 2009; Salter et al., 2010), decided to test opportunities for participatory scenario planning as a component of the NCA3 scenario strategy. The approaches use climate and socioeconomic scenarios to anticipate potential local-scale conditions and impacts, and to explore adaptation (and/or mitigation) options that could address potential impacts and withstand the range of potential future conditions.

An SWG subgroup prepared a short guidance document describing the "scenario planning opportunity for regions and sectors" (Hartmann et al., 2012). The strategy requested that those participating in the NCA, including authors and members of the public who were providing inputs to the process or participating as end users, to: 1) inventory and report on scenario planning activities, including identifying and describing groups using scenario planning; 2) incorporate results of ongoing scenario planning activities into chapters; and 3) undertake a pilot scenario planning activity by working with stakeholders and using a scenario method to conduct the adaptation planning. The guidance document for pilot scenario planning activities encouraged stakeholders to explore adaptation options that address potential risks to natural resources, the economy, or their quality of life. It also asked the participants to use projections

Kenneth Kunkel 7/8/2015 9:55 AM
Deleted: D

Richard Moss 7/26/2015 3:15 PM
Deleted: ,

Richard Moss 7/26/2015 3:16 PM
Deleted: described

Richard Moss 7/26/2015 3:16 PM
Deleted: a

Richard Moss 7/26/2015 3:16 PM
Deleted: y

Richard Moss 7/26/2015 3:16 PM
Deleted: designed to

of potential impacts for their location or sector associated with the B1 and A2 scenarios, drawing on input from the expert science and assessment community. Given these impacts, the next step was to develop narrative scenarios (and if possible, associated quantitative information) on adaptation to these impacts, including specifying needed technologies, financing, institutional developments, and other factors affecting their use. The adaptation scenarios were then to be used as the basis for discussion about the opportunities for implementation, risks, and likely courses of action.

Reflections and issues for evaluation and planning

None of the chapter author teams took up the specific planning scenario suggestions, though the Southwest Regional Climate Assessment Team did document capacity building activities, including scenario planning activities. Unfortunately there were no resources available for widespread dissemination of the scenario planning guidance, or for providing facilitation for those groups that lacked prior experience with participatory scenario techniques. In the rush to complete the report in a timely manner, "the art of the possible" became the rallying cry for the Assessment team, and this particular request did not seem possible in light of imminent deadlines to produce the NCA3 report. This is another issue that will require evaluation, planning, and adequate resources for future assessments.

Kenneth Kunkel 7/8/2015 9:56 AM
Deleted: There is no evidence that any

8. Conclusions and Future Recommendations

Ambitious plans for scenarios resulted in many innovations. We offer several final reflections, evaluation needs, and opportunities that should be considered as part of the sustained assessment process and for future assessment reports.

1. Start earlier: Despite best efforts, the scenario materials developed for NCA3 were delivered to authors piecemeal and late. This made it difficult for them to incorporate in their work. Even in the context of a sustained assessment process, there will be an ongoing challenge because the cycle of production of new climate model simulations and impacts literature based on these simulations does not align with scenario needs for the NCA report requirements. For example, the CMIP activities are typically conducted on the approximate six-year cycle of the IPCC, which is not synchronized with the NCA four-year cycle. The decision to use CMIP3 produced controversy since CMIP5 was being produced at the same time as the NCA3, and several participants felt the old data were built on old scenarios and models and hence not representative of the state of science. Others argued the differences across CMIP cycles were not significant enough to be an issue for use in impact assessment. Paradoxically, the earlier the start, the greater the potential there is to make this aspect of the challenge more problematic for the cyclical assessment process. Given the need for quality control and vetting with users, work on a new cycle would need to begin before the end of an ongoing assessment cycle if results were to be available in time. The analytical capabilities that would be available in a sustained assessment process would not (obviously) address the fundamental structural issues of timing, but they would facilitate much quicker incorporation of new information and data sets.

2. Provide training, not just guidance: A recommendation from evaluations of past NCAs (e.g., Morgan et al., 2005) was to provide guidance and training for assessors. For NCA3, detailed guidance documents were prepared for author teams, and webinars were held to provide training on various topics, including scenarios. A web page of scenario resources was

Kenneth Kunkel 7/24/2015 2:56 PM
Deleted: where it is assumed that scenario materials can be developed in a strategic and timely way,

Kenneth Kunkel 7/24/2015 2:57 PM
Deleted: associated with climate information. T

Kenneth Kunkel 7/24/2015 2:57 PM
Deleted: synthesis

Kenneth Kunkel 7/24/2015 2:58 PM
Deleted: (the implications for 'sustained assessment' are even more complex)

Richard Moss 7/26/2015 3:20 PM
Deleted: Scenarios should be developed, vetted, and tested with developers and users of the scenarios, but in a time frame that enables their use more broadly by other groups involved in the studies. This means that

Richard Moss 7/26/2015 3:20 PM
Deleted: must typically

Richard Moss 7/26/2015 3:20 PM
Deleted: the current

Richard Moss 7/26/2015 3:21 PM
Deleted: are

Richard Moss 7/26/2015 3:21 PM
Deleted: to have impact on analyses

Richard Moss 7/26/2015 3:21 PM
Deleted: Ironically, design of the new assessment and its focus may often influence the design of the scenarios themselves and, thus, such temporal disconnects are at play in both directions.

also prepared (http://scenarios.globalchange.gov). However, based on anecdotal evidence, the guidance documents did not prove sufficient for most users. A particular issue was that they did not address relationships across topics, for example the need for an integrated approach to risk, uncertainty, and scenarios (see Moss, this issue). Additional training opportunities should be provided for both intermediate and end users. For intermediate users, this should include methodologically-oriented knowledge-sharing workshops and mentoring or coaching to illustrate the proper uses and limits of different approaches, including increasing emphasis on the use of socioeconomic scenarios, scenario planning, and risk framing approaches.

3. Develop new, flexible approaches for nesting information within multiple scales and sectors: NCA3 again reverted to fixed, regional boundaries to inform regional chapters rather than to be truly useful for decisions at multiple scales. Although difficult, it should be possible to enable users to define the geographic scope, as already exists in several climate change portals. Focusing exclusively on the large and heterogeneous NCA regions missed opportunities for assessing different spatial and institution scales and for addressing sectoral needs. If the Assessment can consistently "nest" information within various national, regional and local scales, the exact boundaries of the regions become much less important (See NRC 2007, Evaluation of Global Change Assessments, which recommends a "nested" approach). A coordinated effort to truly integrate (not just link to) existing agency portals with current NCA resources may be more cost-effective than building a system from scratch, but it is not trivial. Such a system would further scenarios development that can be adopted and tailored at finer scales with contextual details that illuminate key issues, systems dynamics, and tradeoffs for interested communities.

Kenneth Kunkel 7/24/2015 2:59 PM
Deleted: TSU staff responded to author queries and requests.

Kenneth Kunkel 7/24/2015 3:00 PM
Deleted: on using and interpreting scenarios

Kenneth Kunkel 7/24/2015 3:02 PM
Deleted: . Information was prepared

Kenneth Kunkel 7/27/2015 8:02 PM
Deleted: it is perhaps impractical to develop fine-scale scenarios for every locale

Kenneth Kunkel 7/27/2015 8:03 PM
Deleted: tailored for every application,

Kenneth Kunkel 7/24/2015 3:03 PM
Deleted: deploy information in a way that

Kenneth Kunkel 7/24/2015 3:03 PM
Deleted: s

Kenneth Kunkel 7/24/2015 3:04 PM
Deleted: The NCA regions are large and heterogeneous, and f

Richard Moss 7/26/2015 3:24 PM
Deleted:

Kenneth Kunkel 7/24/2015 3:04 PM
Deleted: m

Richard Moss 7/26/2015 3:24 PM
Deleted: e

Richard Moss 7/26/2015 3:24 PM
Deleted: n

Kenneth Kunkel 7/27/2015 8:04 PM
Deleted: to achieve a user-friendly outcome

Kenneth Kunkel 7/27/2015 8:04 PM
Deleted: serve as a springboard for

4. Provide ongoing engagement and advice from both the scientific and end user communities in scenario development: Schedules and requirements within agencies supporting the NCA, as well as for lead authors and others likely to be involved in preparing future reports, require that preparations for the next assessment begin as soon as the last one is complete. It is essential to engage the communities involved in the sustained assessment and specific reports to ensure that past efforts are evaluated, needs are clearly specified, and the potential for innovations explored. Federal agencies should assemble an informal community of scenario practice or a more formal advisory body to serve as a sounding board and to develop recommendations on how to best provide scenarios and other information.

5. Ensure consistency of scenarios: A further challenge is capacity for combining domain-specific scenarios (e.g., climate change, sea level rise, population/demographics, land use and land cover change, technology evolution, etc.) into integrated scenarios that are consistent and plausible across domains. An interagency group (Scenarios and Interpretive Science Coordinating Group) within the USGCRP has undertaken efforts to improve scenarios with intentions to: 1) advance collaborative science on critical gaps; 2) enhance methodologies for use-inspired scenario development, risk framing, and contextual interpretation; 3) develop the next generation scenario work products for model intercomparisons, assessments, and analyses; and 4) improve interagency communications, coordination, and accessibility to knowledge, work products, and technical resources.

The innovations in scenario information for the NCA3 were intended to provide the foundation for a rich and cutting-edge assessment. Not all expectations could be met, however, and the process highlighted other needs to be addressed in the future. A sustained assessment

Kenneth Kunkel 7/24/2015 3:06 PM
Deleted: in developing meaningful scenarios

Richard Moss 7/26/2015 3:26 PM
Deleted: advances

Richard Moss 7/26/2015 3:27 PM
Deleted: the characteristics of

Richard Moss 7/26/2015 3:26 PM
Deleted: s

Richard Moss 7/26/2015 3:27 PM
Deleted: the development of

Richard Moss 7/26/2015 3:27 PM
Deleted: considerable and provided

Richard Moss 7/26/2015 3:28 PM
Deleted: However, the advances in some areas raised

Richard Moss 7/26/2015 3:28 PM
Deleted: that could not be met

process that evaluates past experience and addresses areas of unmet need will improve scenarios for future assessments and other applications.

Richard Moss 7/26/2015 3:29 PM
Deleted: can work on these

Richard Moss 7/26/2015 3:29 PM
Deleted: between assessments

9. Acknowledgements

This work was partially supported by NOAA through the Cooperative Institute for Climate and Satellites — North Carolina under Cooperative Agreement NA09NES4400006 and by the NOAA Climate Program Office, Climate Observations and Monitoring Program. The DOE Office of Science supports basic research on scenarios and supported several workshops that reviewed the state of science in scenarios and related areas of modeling. Robert Vallario provided very helpful insights on these and related aspects of the NCA3 scenarios development process. NASA provided support for the NCA scenario development process.
